# Supplementary material for: Many-body interference in kagome crystals
Source: Nature. 2025 Oct 29;647(8088):68–73. doi: 10.1038/s41586-025-09659-8 (PMC12589133; doi:10.1038/s41586-025-09659-8)
Supplement: Supplementary file 1 — The supplementary information contains eight sections, 16 display items and references [file 41586_2025_9659_MOESM1_ESM.pdf]

nature portfolio

https://doi.org/10.1038/s41586-025-09659-8

Supplementary information

Many-body interference in kagome crystals

In the format provided by the
authors and unedited

Supplementary materials for ”Many-body interference in Kagome crystals”

Chunyu (Mark) Guo†,[1] Kaize Wang,[1] Ling Zhang,[1] Carsten Putzke,[1] Dong Chen,[2,3] Maarten R. van Delft,[4,5] Steffen Wiedmann,[4,5] Fedor F. Balakirev,[6] Ross D. McDonald,[6] Martin Gutierrez-Amigo,[7] Manex Alkorta,[8,9] Ion Errea,[8,9,10] Maia G. Vergniory,[10,11] Takashi Oka,[12] Roderich Moessner,[13] Mark H. Fischer,[14] Titus Neupert,[14] Claudia Felser,[3] and Philip J. W. Moll†[1]

[1]*Max Planck Institute for the Structure and Dynamics of Matter, Hamburg, Germany*

[2]*College of Physics, Qingdao University, Qingdao, China*

[3]*Max Planck Institute for Chemical Physics of Solids, Dresden, Germany*

[4]*High Field Magnet Laboratory (HFML - EMFL), Radboud University, Toernooiveld 7, 6525 ED Nijmegen, The Netherlands*

[5]*Radboud University, Institute for Molecules and Materials, Nijmegen 6525 AJ, Netherlands*

[6]*National High Magnetic Field Laboratory, Los Alamos National Laboratory, Los Alamos, New Mexico 87545, USA*

[7]*Department of Applied Physics, Aalto University School of Science, FI-00076 Aalto, Finland*

[8]*Centro de Física de Materiales (CFM-MPC), CSIC-UPV/EHU, Donostia-San Sebastian, Spain*

[9]*Fisika Aplikatua Saila, Gipuzkoako Ingeniaritza Eskola, University of the Basque Country (UPV/EHU), Donostia-San Sebastian, Spain*

[10]*Donostia International Physics Center, Donostia-San Sebastian, Spain*

[11]*Département de Physique et Institut Quantique, Université de Sherbrooke, Sherbrooke QC J1K 2R1, Canada*

[12]*The Institute for Solid State Physics, The University of Tokyo, Kashiwa, Japan*

[13]*Max Planck Institute for the Physics of Complex Systems, Dresden, Germany*

[14]*Department of Physics, University of Zürich, Zürich, Switzerland*

(Dated: September 12, 2025)

†Corresponding authors: chunyu.guo@mpsd.mpg.de(C.G.); philip.moll@mpsd.mpg.de(P.J.W.M.).

A. Transport mean free path analysis

The transport mean free path l_t denotes the distance an excited quasiparticle travels before its momentum has been randomized by scattering. The idea is based on the decay time of a DC transport current when the driving DC electric field is instantaneously removed at some point in time. Scattering processes with the lattice and its defects will transfer momentum from the electron system and eventually bring it to rest, leading to a characteristic current decay $I(t) = I_0 e^{-t/\tau_t}$. This transport lifetime τ_t can be translated to a mean distance between scattering events of a quasiparticle at the speed of the Fermi velocity v_F simply via $l_t = v_F \tau_t$, which can be related to conductivities via Boltzmann transport. Such a picture of freely traveling quasiparticles between scattering events is critical in mesoscopic physics when device sizes are similar to this mean distance between scattering. However, this simple logic only rigorously works in simple cases, in particular in isotropic metals (spherical Fermi surface) with only one single band and carrier type. For complex multi-band metals hosting both electrons and holes on Fermi surface sheets with non-spherical shapes, this assumption is not generally valid. Scattering processes are momentum- and band-dependent, and a global decay time cannot be assumed to reflect directly onto each individual electron state. On top, the strong electron-phonon coupling results in non-trivial momentum dependences of the scattering matrix elements via the phonon-dispersion. As a result, the question of ballistic transport in such materials, in practice, is challenging. The goal is to apply the simplest analysis and show its mean free path to fall substantially below the device size, hence speaking against ballistic transport.

We start by estimating the transport mean free path based on the Drude model:

$$\sigma_{xx} = ne^2\tau/m^* \tag{S1}$$

The conductivity is directly obtained from the measurements of in-plane resistivity of device S5. The carrier density is extracted from the previous report of Hall voltage measurements on CsV_3Sb_5[1]. At T = 5 K, the hall resistivity ρ_{xy} reaches 0.47 $\mu\Omega$ cm at B = 14 T. Given the almost linear-in-field ρ_{xy}, the carrier density n is estimated to be ≈ 1.86 x 10^{22} cm^{-3} under the assumption of single conduction band. For simplicity, we also assume that the carrier density is temperature-independent, as the temperature variation of Hall resistivity is mainly attributed to the evolution of

carrier mobilities of different pockets while the electronic structure remains almost intact below 50 K. Since the Brillouin zone is majorly occupied by the hexagonal Fermi surface, the in-plane Fermi wavevector can be estimated by: $n = \frac{3\sqrt{3}}{2} k_{F,x}^2 k_{F,z}/4\pi^3$ with $k_{F,z}$ directly determined by the lattice constant $c \approx 9$ Å ($k_{F,z} = 2\pi/c$). Therefore, the transport mean free path can be calculated by:

$$l_t = v_F \tau = \frac{\hbar k_{F,x} \sigma}{ne^2} = \frac{\hbar \sigma}{e^2 \sqrt{0.1316n/c}} \tag{S2}$$

which allows us to extract the transport mean free path directly from the temperature dependence of resistivity (Fig. S 1). Right above the superconducting transition temperature (T_c = 2.8 K), the transport mean free path reaches 500 nm and then quickly drops to 150 nm as the temperature increases to 20 K. These values are well below the characteristic sizes of all devices measured.

As stated above, this estimate is based on crude approximations. Firstly, due to the slightly non-linear field dependence of Hall resistivity, the single-band assumption may lead to an underestimation of the total carrier count across multiple conduction bands. Secondly, the assumption of a singular, hexagonal 2D Fermi surface oversimplifies the complex Fermiology in CsV_3Sb_5, which affects the approximation of the Fermi wavelength based on carrier density. As the single-band assumption tends to overestimate the averaged Fermi velocity, these estimations are rather an upper bound for the transport mean free path l_t. Given the aforementioned difficulties, one may still argue for substantially suppressed scattering for some regions of one of the Fermi surfaces to reach a quasi-ballistic limit for these few select states only. However, this would imply an extreme variation of lifetime across the Fermi surface, a highly unusual feature of materials dominated by impurity scattering.

Given these apparent difficulties in simple Drude analysis, we also explored the size-dependent resistivity in CsV_3Sb_5(Fig. S 2). A series of in-plane microstructures with varying sizes is fabricated and measured down to the base temperature. At room temperature, the resistivity among different devices remains almost identical to the bulk resistivity value, demonstrating an unchanged crystal quality after FIB fabrication processes. The residual resistivity at low temperature, however, displays a visible increase with decreasing sample size. This change is naturally attributed to the boundary scattering enhancement with the characteristic sample size approaching the transport mean free path. Importantly, at the smallest sample size measured, $W = 1.1\mu$m, the residual resistivity increased about 25% compared to its bulk value. This marginal increase suggests that l_t is substantially shorter than 1.1μm. By fitting the size dependence of residual resistivity using

an isotropic ballistic model[2], the mean free path l_t is estimated to be approximately 560 nm. This value is consistent with the analysis of the Drude model, which further supports our conclusion that the sizes of the microstructures we studied are all substantially larger than l_t, especially at elevated temperatures. These results highlight the significance of collective electron behavior in CsV_3Sb_5 that extends well beyond a single-particle ballistic transport scenario.

B. Dingle analysis for extracting quantum mean free path

The quantum mean free path is obtained by analyzing the field dependence of the SdH oscillation amplitude measured with the magnetic field applied along the c-direction (Fig. S 3). According to the Lifshitz-Kosevich model, the oscillations can be described as[3]:

$$\Delta\rho_{\|}/\rho_{\|}^{0} \propto \sqrt{B}\sum_{r=1}^{\infty}\frac{R_D^r R_T^r}{r^{1/2}}\cos(r\lambda)\cos\left[r\left(2\pi\frac{F_0}{B}+\pi\right)+\phi_{LK}\right]. \tag{S3}$$

The oscillation amplitude can be smeared due to the finite quasiparticle lifetime τ and nonzero temperature T, which result in the well-known Dingle amplitude factor and thermal damping factor, respectively:

$$R_D^r := \exp\left(-\frac{r}{2}\frac{h}{\varepsilon_c\tau_q}\right), \quad R_T^r := \frac{a_r}{\sinh\left(a_r\right)}, \quad a_r := \frac{2\pi^2 r k_B T}{\varepsilon_c}, \tag{S4}$$

with $\varepsilon_c = \hbar|eB|/m_c$ the cyclotron energy, $m_c = (2\pi)^{-1}\partial S/\partial E$ the cyclotron mass, and τ_q the quantum lifetime. We first determine m_c via the temperature dependence of oscillation amplitude (Fig. S 3b), while the exponential growth of oscillation amplitude with increasing magnetic field stands for the enlarging ratio between the cyclotron energy and the Landau level broadening due to scattering events and therefore can be used to obtain the quantum lifetime. The relation between the field dependence of quantum oscillation amplitude and quantum life time τ_q can be expressed as:

$$f(B^{-1}) = \ln[Amp\sqrt{B}\sinh(14.69 m_c T/B)] = -\frac{\pi m_c}{e\tau_q}B^{-1} + Const. \tag{S5}$$

by extracting the linear slope of the $f(B^{-1})$(Fig. S 4), τ_q can be obtained. Its inaccuracy is determined by the uncertainty of the linear fitting (see error bars in Fig. 3). The calculation of the quantum mean free path, $l_q = v_F\tau_q$, further requires the value of Fermi velocity v_F, which can be directly calculated with the oscillation frequency and cyclotron mass:

$$v_F = \frac{\hbar k_F}{m_c} = \frac{\sqrt{2\hbar e F}}{m_c} \tag{S6}$$

This allows us to determine the temperature dependence of l_q for both the γ and δ pockets as shown in Fig. 3a.

C. Observation of h/e oscillations in low-quality crystals

To further demonstrate the non-trivial origin of h/e oscillations, we have fabricated additional microstructures using a comparatively low-quality crystal(Fig. S 5). This difference between crystals is mainly controlled by the purity of the starting materials used in the flux-growth process. As suggested by the clear increase of residual resistivity by more than 250%, the scattering rate is substantially enhanced in this crystal. Unexpectedly, the h/e oscillations, despite the reduction in amplitude, remain visible in this microstructure. This is an important observation since the sample width now exceeds ten times the transport mean free path, at which condition the h/e oscillations in $PdCoO_2$ are no longer observable[4]. Consistently, the overall width dependence of oscillation amplitude displays a much slower suppression than the expectation of a single-particle scenario (Fig. S 6), where the amplitude decreases exponentially with increasing width. These results provide crucial evidence for the long-range quantum coherence of collective electrons in CsV_3Sb_5.

D. Comparison between h/e oscillations and chiral transport

As illustrated in Fig. 3b, the amplitude of the h/e oscillations is strongly suppressed when the magnetic field is rotated away from the in-plane direction. Such a distinct angular dependence directly corresponds to the magneto-chiral transport as previously observed[5]. The absolute value of the second harmonic voltage $V_{2\omega}$ signal, which stands for the non-reciprocal transport signature in CsV_3Sb_5, displays an identical angular dependence with the h/e oscillations (Fig. S 7). This striking correspondence is consistently observed in both devices S2 and S4, suggesting the significant modulation of electronic orders in CsV_3Sb_5 and the possible role of quantum phase coherence in the appearance of the chiral-magneto transport.

E. Simulation of semiclassical Bloch-Lorentz oscillations

Here, we present the formalism for the semiclassical B-periodic oscillation. We use the Boltzmann transport equation(BTE) to calculate the magneto-conductivity σ_{zz} along c-axis of finite-size quasi-two-dimensional materials.

For quasi-two-dimensional materials, the band dispersion is modeled by

$$\varepsilon(\mathbf{k}) = \varepsilon_{\parallel}(\mathbf{k}_{\parallel}) - t_z \cos(k_z d), \tag{S7}$$

where $\mathbf{k}_{\parallel} = (k_x, k_y)$ is the in-plane momentum and d is the interlayer distance. The out-of-plane coupling t_z is assumed to be weak, i.e., t_z is much smaller than the bandwidth given by $\varepsilon_{\parallel}$.

We use linearized ansatz for the distribution function $f(\mathbf{r}, \mathbf{k})$,

$$f(\mathbf{r}, \mathbf{k}) = f^0 - \frac{\partial f^0}{\partial \varepsilon} h(\mathbf{r}, \mathbf{k}), \tag{S8}$$

where f^0 is the Fermi-Dirac distribution function.

Assuming we impose an electric filed $\mathcal{E}_z$ in the z-direction, the BTE under relaxation time approximation reads

$$\mathbf{v}(\mathbf{k}) \cdot \nabla_{\mathbf{r}} h - \frac{e}{\hbar}[\mathbf{v}(\mathbf{k}) \times \mathbf{B}] \cdot \nabla_{\mathbf{k}} h - e v_z(k_z)\mathcal{E}_z = -\frac{h(\mathbf{r}, \mathbf{k})}{\tau}, \tag{S9}$$

where $\mathbf{v}(k) = \partial\varepsilon(\mathbf{k})/\partial\mathbf{k}$ is Fermi velocity vector and $\mathbf{B} \equiv B(\cos\theta\cos\varphi, \cos\theta\sin\varphi, \sin\theta)$ is external magnetic field.

For a finite-size sample with length L and width w, we impose a completely diffusive boundary condition,

$$h(\mathbf{r}_B, \mathbf{k}_{\parallel}, k_z) = 0 \qquad \mathbf{v}(\mathbf{k}_{\parallel}) \cdot \hat{\mathbf{n}}_B < 0, \tag{S10}$$

where $\mathbf{r}_B$ is arbitrary position vector at the boundary and $\hat{\mathbf{n}}_B$ is the norm vector at $\mathbf{r}_B$.

With this boundary condition, the distribution function $h(\mathbf{r}, \mathbf{k})$ can be exactly solved by the method of characteristics[6,7]. Therefore, the conductivity can be calculated as

$$\sigma_{zz} = \frac{e}{S_{\parallel}\mathcal{E}_z} \int_{S_{\parallel}} d^2\mathbf{r}_{\parallel} \int_{\mathrm{BZ}} \delta(\varepsilon - \varepsilon_F) h(\mathbf{r}, \mathbf{k}) v_z(\mathbf{k}) d^3k \tag{S11}$$

For an out-of-plane magnetic field with moderate tilting angle θ up to 30°, we use an isotropic dispersion $\varepsilon_{\parallel}(\mathbf{k}_{\parallel}) = {\mathbf{k}_{\parallel}}^2/2m^*$ to model the in-plane dispersion. The solution gives rise to B-periodic oscillation of the conductivity as shown in Fig. S 8. The physical reason for this is that

when the oscillation period is commensurate with the sample width, all the semiclassical trajectories have a zero net z-direction displacement over the time of flight. Resulting in a minimal at every commensurate field[6]. Since a moderate tilting angle θ does not change the in-plane trajectory drastically, the commensurate condition still holds except for a pre-factor $\cos\theta$ due to projection. Thus, the semiclassical B-period oscillation can be observed over a large window of out-of-plane angle θ. To elaborate further on the experimental conditions, we have also simulated the Bloch-Lorentz oscillations based on realistic Fermi surfaces identified in DFT calculations. These oscillations are consistently visible up to at least 15 degrees, which contrasts with the experimental results(Fig. S 9).

We also present the result of varying φ at $\theta = 0°$ for the in-plane magnetic field. We try two different models for the in-plane dispersion $\varepsilon_{\parallel}$: cylindrical and hexagonal models (Fig. S 10). Surprisingly, when rotating away from the principal axis, the oscillation vanishes quickly for a cylindrical Fermi surface. On the other hand, the oscillation remains visible when rotating to a larger angle, as in the case of a hexagonal Fermi surface. Yet, multiple oscillation frequencies coexist due to the three dominant directions of Fermi velocities. Therefore, the simulation in both limiting cases cannot capture the experimental signatures even qualitatively, particularly regarding the angular dependence.

The most prominent difference between our observations and semiclassical models lies in the thickness (D) dependence of these oscillations. In Bloch-Lorentz simulations, the oscillations can be significantly suppressed when the device thickness is comparable to the magnetic length (Fig. S 11); therefore, the oscillation amplitude drops monotonically with decreasing thickness-to-width ratio (D/W). This is, however, in stark contrast with our experimental observations. Across three devices with similar width yet distinct thickness, the oscillations remain almost constant (Fig. S 12). This comparison provided key evidence for the failure of the semiclassical picture in describing the h/e oscillations observed in CsV_3Sb_5.

F. Consistent switch of oscillation periods

To investigate the universality of the switching behavior illustrated in Fig. 2, we measured the angular dependence of oscillation periodicity in three microstructures. Devices S1 and S4 have significantly different dimensions at their cross-sections. Device S1 features a narrow channel, with its width being nearly half its depth and, therefore, distinct oscillation periods and amplitudes

with the field applied either parallel or perpendicular to the crystalline a-direction (Fig. S 13a-d). Meanwhile, such a difference is much less significant in S4 due to its comparable width and depth (Fig. S 13e-g). Despite their distinction in dimensions, the periodicity switch at 45 degrees modulo 90 degrees was consistently observed in both samples (Fig. S 14). To explore the nature of the consistent switching observed in both devices, we fabricated the cross-section of device S3 into a parallelogram possessing interior angles of $60°$ and $120°$ to match the symmetry of the Kagome lattice. In this case, while the large oscillations again follow the same $\cos(\varphi)^{-1}$ scaling, set by the different widths of both sidewalls. The results demonstrate that the shape is a key factor in determining the switching angles(Fig. S 15), as in both cases, the switching angles match the internal angles of the geometry (rectangle: $90°$; parallelogram: $60°$, $120°$). These results consistently emphasize the irrelevance of the device geometry and its close connection to the correlated electronic order.

G. Temperature dependence across various devices

As microstructures vary in size, the reduction of h/e oscillation amplitude with increasing temperature behaves differently across various devices. Reducing the device's width shifts the onset of the oscillations, defined by the temperature where the oscillation amplitude gets larger than 5% of the amplitude at T = 2 K, up to a higher temperature (Fig. S 16). By linearly extrapolating to zero width, the onset temperature is determined to be about 33 K, which coincides with the T' as explained. It is much higher than the onset of quantum oscillations in CsV_3Sb_5 (Fig. S 3), which displays no variation across all devices of different sizes.

H. Theoretical interpretation

Our unexpected findings remain at present theoretically unexplained, yet they are of natural importance for our understanding of CsV_3Sb_5, Kagome materials more generally, and quantum coherence in solids at large. While the appearance of an apparent coherence limited by sample size is a spectacular phenomenon in and of its own, the present experiment fundamentally prompts a question which may be phrased in the form of a paradox: how can the absence of well-resolved quantum oscillations be reconciled with the magnetoconductance oscillations, given the fact that coherence lengths extracted in conventional ways for both phenomena are hugely incompatible?

Indeed, a hint toward an explanation stems from the temperature dependence (Main Fig.4). The amplitudes remain unaffected until about 10 K, above which they decay into the noise floor around 30 K. This temperature dependence quantitatively agrees with that of multiple electronic probes (Fig. 4b), including STM[8], μSR[9], NMR[10], anomalous Nernst effect[11], electrical transport[12–14] and magnetochiral effects[5]. Further, the strong influence of the out-of-plane field component quantitatively mirrors field-switchable diode effects measured at much higher values of the in-plane field (see supplement) and chimes in the choir of experimental evidence showing field-switchability by out-of-plane fields, including STM[15], non-reciprocal transport[5], electronic anisotropy[12] and magneto-optical Kerr effect[16]. This remarkable agreement strongly suggests a substantial change in the electronic spectrum, which now appears to host quantum coherence on the micron scale. Positing the appearance of an emergent order as being instrumental, the following further scenarios present themselves.

One is that the phase coherence is a 'parasitic' effect, feeding off the stability of another phase variable. For instance, a CDW pattern may conceivably imprint its phase on the conduction electrons carrying charge between different layers. Such a phase degree of freedom may perhaps be modeled as a bosonic mode that extends across the sample thanks to symmetry breaking/long-range order. Such a theory would still need to be supplemented by an analysis showing that, in this scenario, quantum oscillations are not stabilized also.

Another viewpoint may be that the gap opening due to the emergent order has a strong effect on the actual coherence length. Given that scattering processes can be very non-uniform across hexagonal Fermi surfaces incorporating non-trivial orbital wavefunction content[17], the presence of such an effective suppression of scattering hotspots is quite plausible. Its size—extending coherence lengths to the macroscopic realm as suggested in our experiment—would, however, still be astonishing.

A further, conceptually particularly clean picture would emerge in a limiting case in which the quantum oscillations and the magnetotransport oscillations are ascribed to different degrees of freedom which only weakly intercommunicate. This again is not implausible given the complexity of the multi-component Fermi surface, separated in a cylindrical Sb p-orbital pocket and multiple near-hexagonal V d-orbital pockets. The charge order is known to reconstruct the V pockets, and to reduce the corresponding density of states, while it has a much weaker effect on the Sb pocket. The current sustaining the oscillations would then be transported by the Sb p band. With a nearly isotropic pocket, one would expect the in-plane angle dependence of the oscillations to

be dominated by sample geometry rather than lattice effects, in line with the observations. Such an "orbital decoupling" scenario is also in line with the recent experiments on superconductivity in CsV_3Sb_5, where a nearly independent superconducting gap formation at different temperatures was observed on the d and p pockets[18,19].

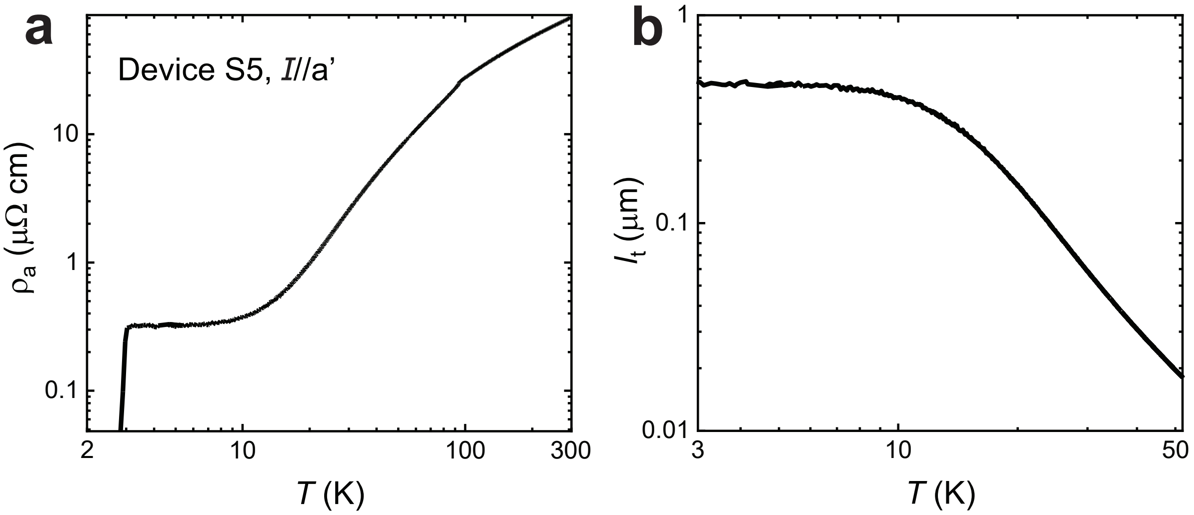

Fig. S 1. **Transport mean free path extracted from Drude model.** (a) In-plane resistivity measured with current applied perpendicular to the a-direction. The kink at 94 K stands for the charge-density-wave transition, while the superconducting transition is at T_c = 2.8 K. Both values agree well with the previous report on bulk samples[11,20], demonstrating the unchanged device properties after FIB-fabrication. (b) Transport mean free path estimated from the in-plane resistivity based on the Drude model.

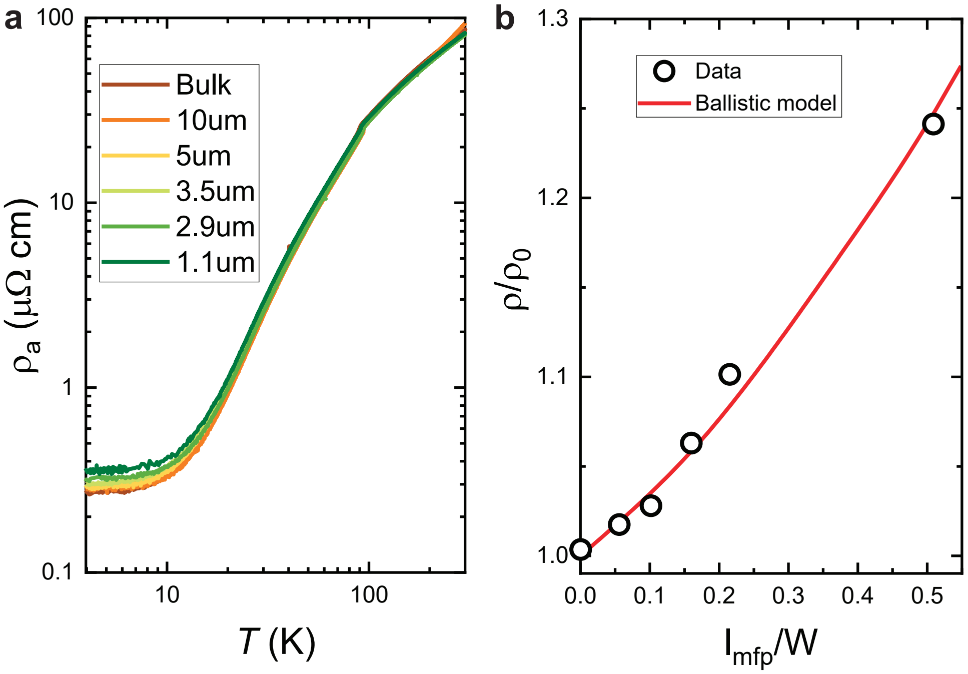

Fig. S 2. **Size-scaling of residual resistivity.** (a) Temperature dependence of in-plane resistivity at various sizes. The resistivity at $T = 4$ K is monotonically increasing with decreasing sample size, demonstrating the enhancement of boundary scattering when the sample size approaches the transport mean free path of CsV_3Sb_5. (b) Size-dependent residual resistivity displays a consistent scaling with the ballistic transport model described in previous reports of $PdCoO_2$[2]. This analysis yields a transport mean free path of about 560 nm at base temperature, which is consistent with the estimation based on the simple Drude model.

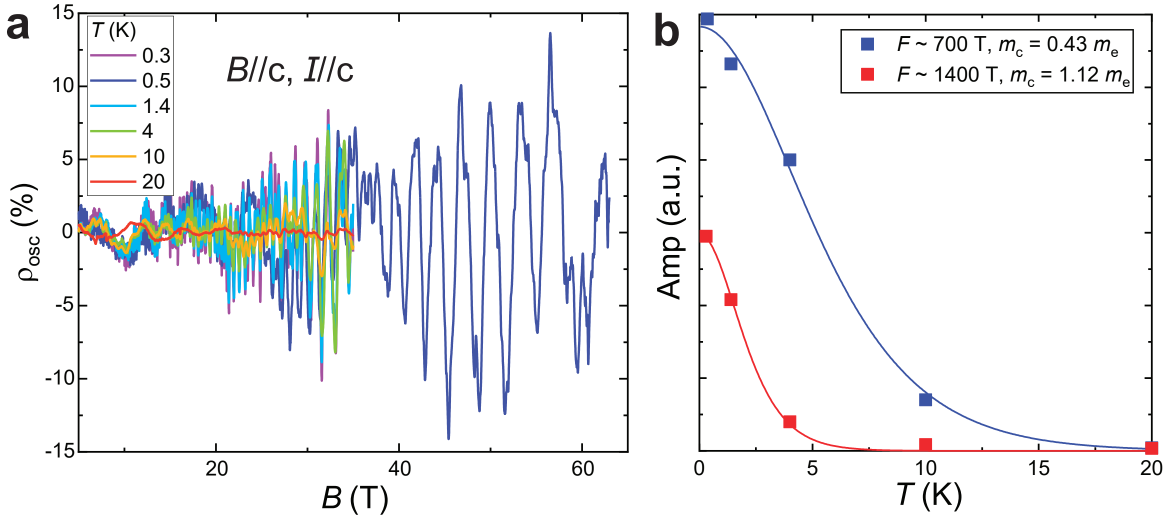

Fig. S 3. **Quantum oscillations and cyclotron mass analysis.** (a) Quantum oscillations at various temperatures. The oscillations are obtained from the magnetoresistance by subtracting a 5th-order polynomial fitting as a background. The measurement at T = 0.5 K is performed in a pulsed magnet up to 63 T, while others are measured in a water-cooled static magnet up to 35 T. (b) Lifshitz-Kosevich fitting to the temperature dependence of oscillation amplitudes determines the cyclotron masses of two main Fermi pockets in CsV_3Sb_5.

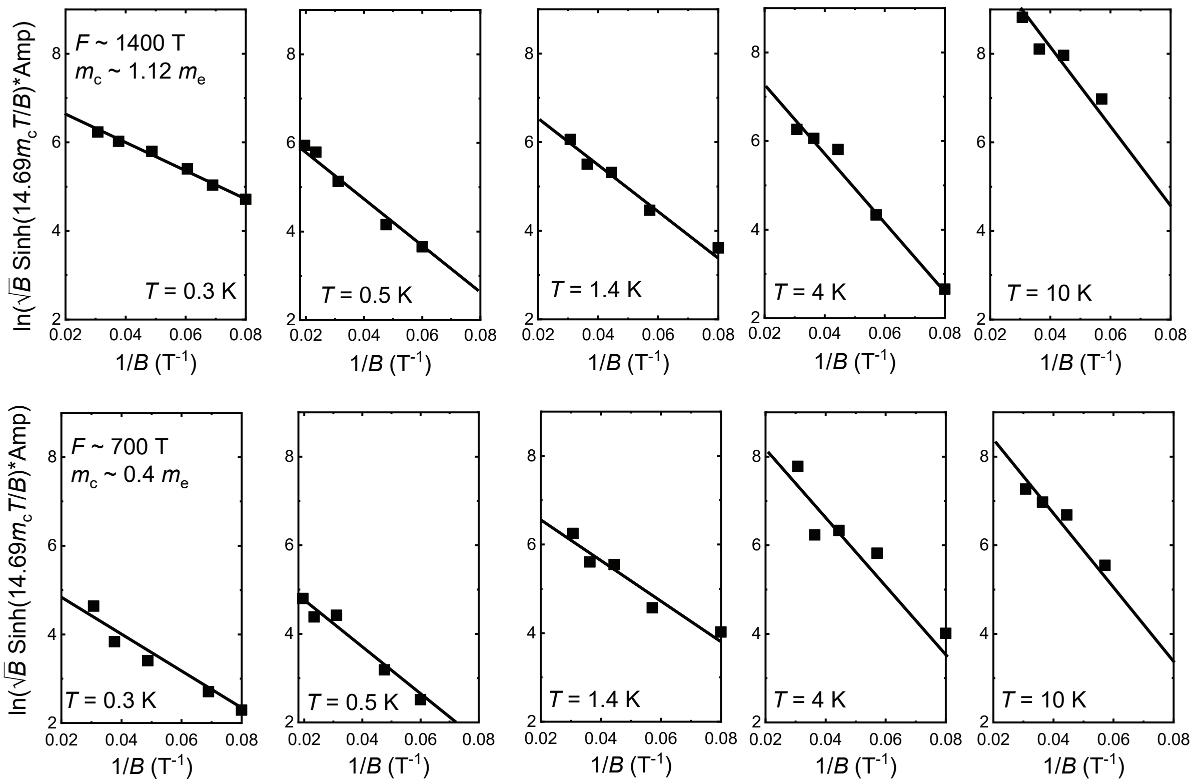

Fig. S 4. **Quantum mean free path extracted from Dingle analysis.** Extrapolation of field-dependent oscillation amplitude yields the quantum lifetime for each Fermi surface at various temperatures. The linear fitting coefficient leads to the determination of the quantum mean free path as presented in Fig. 3a.

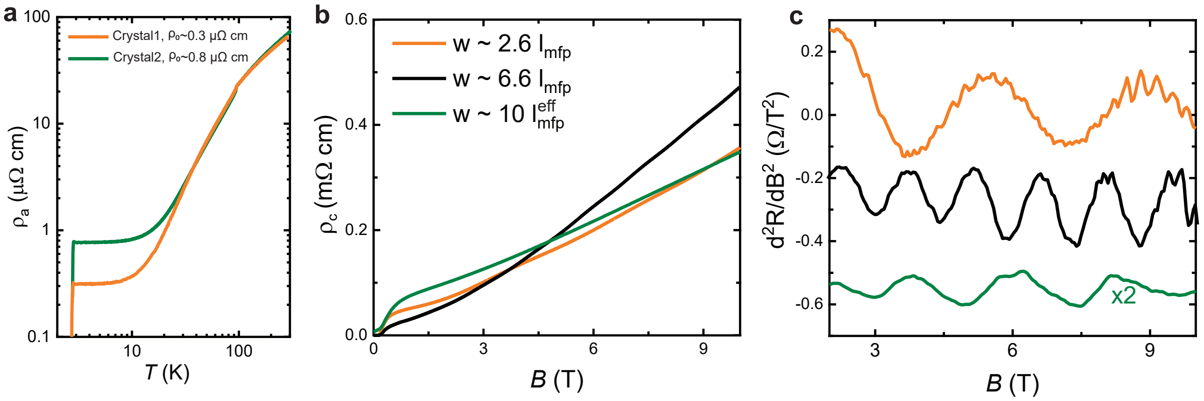

Fig. S 5. h/e **oscillations observed in low-quality samples.** (a) Comparison of temperature-dependent resistivity between high-quality sample (crystal 1) and low-quality sample (crystal 2). The residual resistivity is enhanced 2.5 times in the low-quality crystal, demonstrating a substantial increase in the electron scattering rate. (b) and (c) present the magnetoresistivity and extracted h/e oscillations among different devices, respectively. The oscillations remain visible even when the sample width is significantly larger than its transport mean free path. This clearly distinguishes from the h/e oscillations observed in $PdCoO_2$ and, therefore, rules out ballistic transport as its origin.

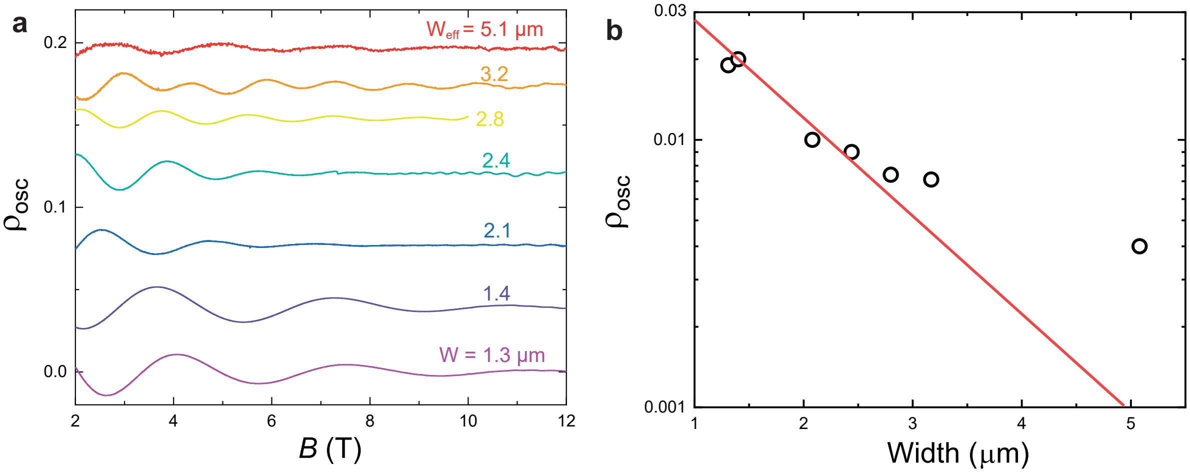

Fig. S 6. **Scaling of oscillation amplitude versus sample width.** (a) h/e oscillations observed among various devices with different widths. Here ρ_{osc} stands for the rescaled oscillatory magnetoresistivity, ρ_{osc} = $\Delta\rho/\rho_{bg}$, with $\Delta\rho$ the subtracted oscillatory part, and ρ_{bg} the 5th-order polynomial background used for the subtraction. (b) The width dependence of ρ_{osc} demonstrates a much slower decay than simple exponential scaling, indicating its non-trivial origin other than ballistic transport.

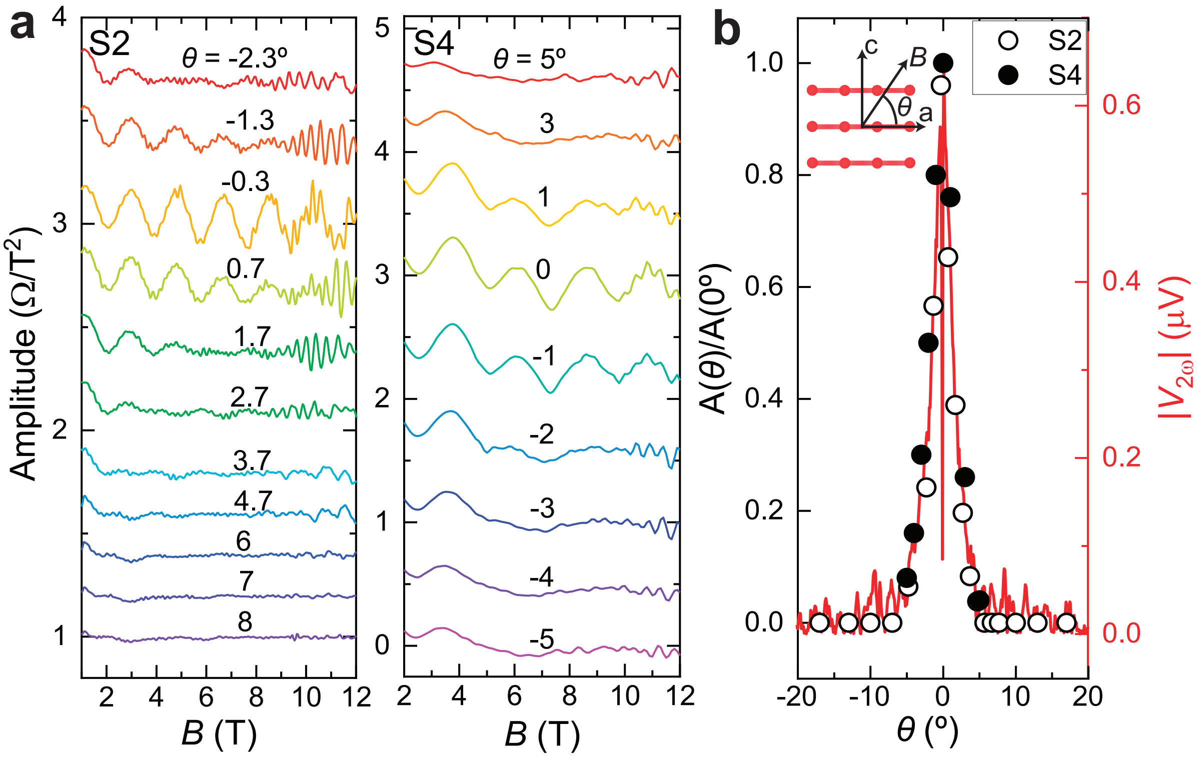

Fig. S 7. **Identical angular dependence of chiral magneto transport and h/e oscillations.** (a) h/e oscillations measured at different out-of-plane angle θ for both device S2 and S4. The oscillation becomes almost invisible at angles larger than 5°. (b) Comparison between h/e oscillations and the non-reciprocal transport signature $V_{2\omega}$[5]. Their identical angular dependence suggests the close relation between them.

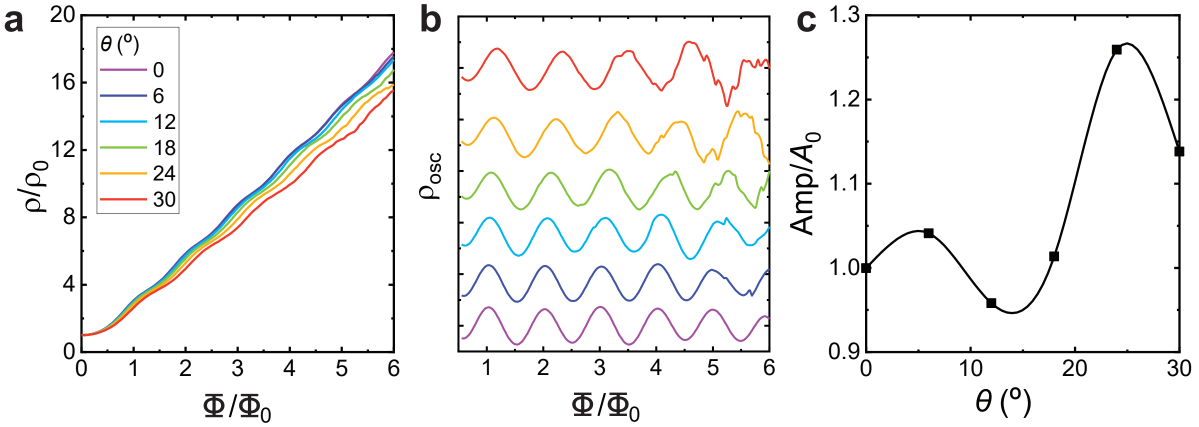

Fig. S 8. **Angular dependence of simulated Bloch-Lorentz oscillations based on a cylindrical Fermi surface.** (a) and (b) stands for the simulated magnetoresistance and Bloch-Lorentz oscillations, respectively. With the magnetic field rotated from in-plane to out-of-plane direction, the oscillation amplitude merely changes slightly, as shown in (c), which is distinct from the experimental observations.

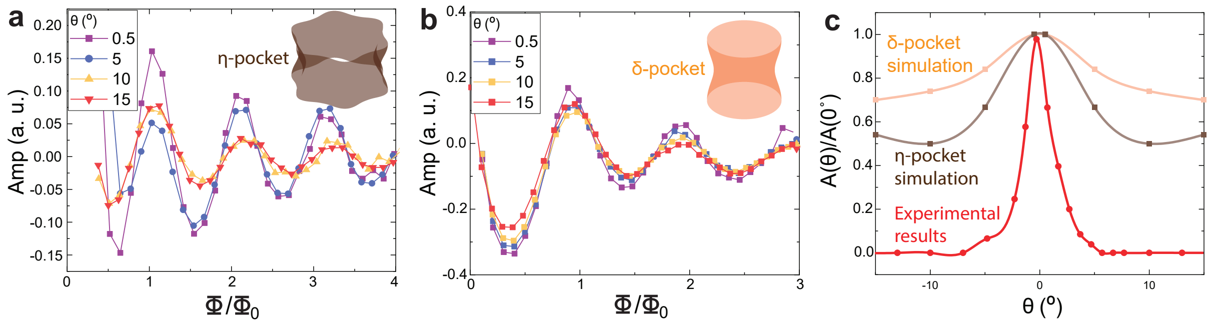

Fig. S 9. **Angular dependence of simulated Bloch-Lorentz oscillations based on realistic Fermi surfaces.** (a) and (b) stand for the simulated Bloch-Lorentz oscillations based on two different Fermi surfaces, respectively. The distinction from experimental results is consistently captured as shown in (c).

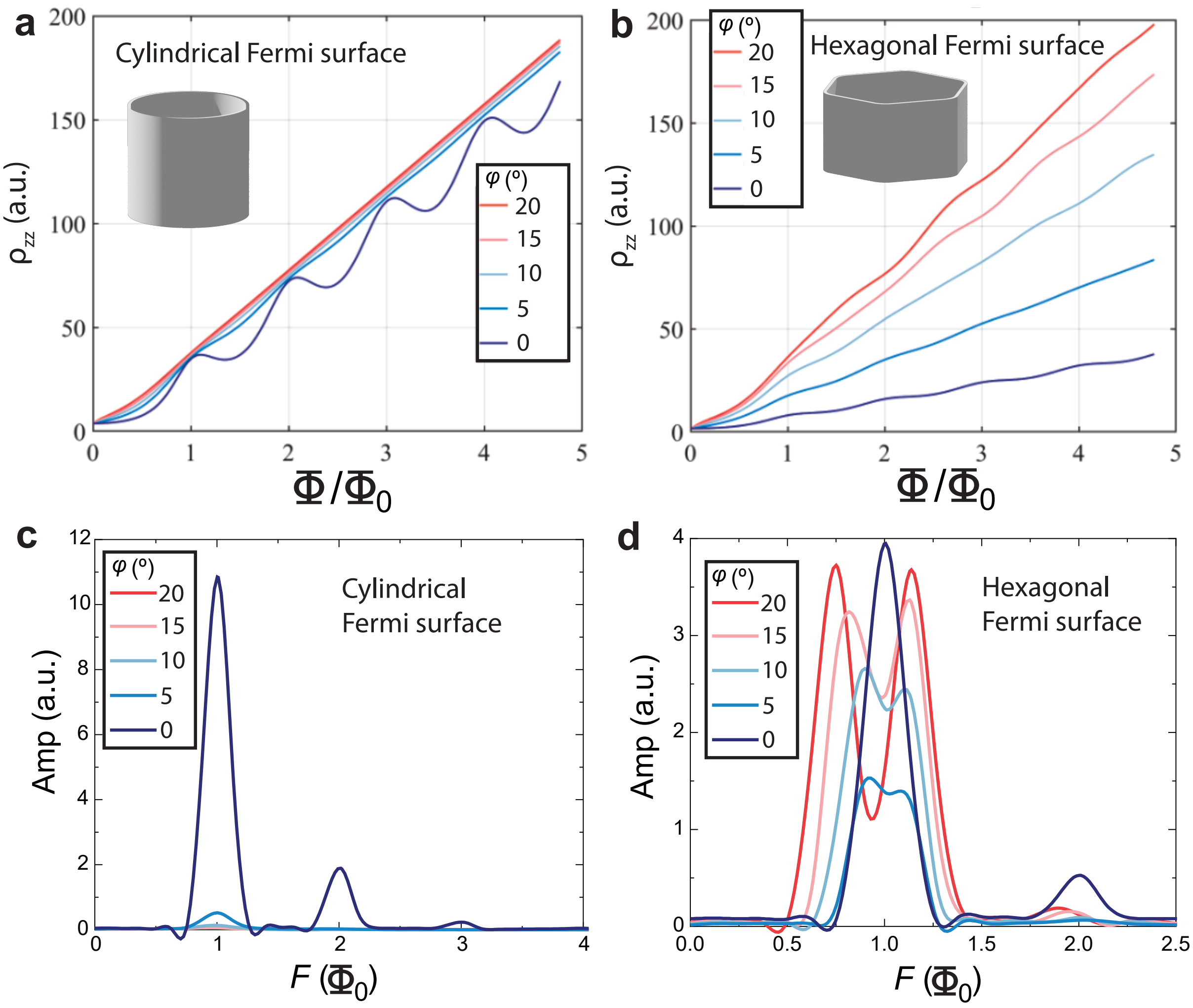

Fig. S 10. **Comparison between cylindrical and hexagonal Fermi surface.** (a) and (b) stand for the Bloch-Lorentz oscillation simulation for cylindrical and hexagonal Fermi surfaces respectively, while (c) and (d) present the FFT analysis of the oscillatory parts in both cases. With the magnetic field rotated in-plane, the oscillation amplitude varies quickly in the case of cylindrical Fermi surface, while its change in the hexagonal case is not as significant.

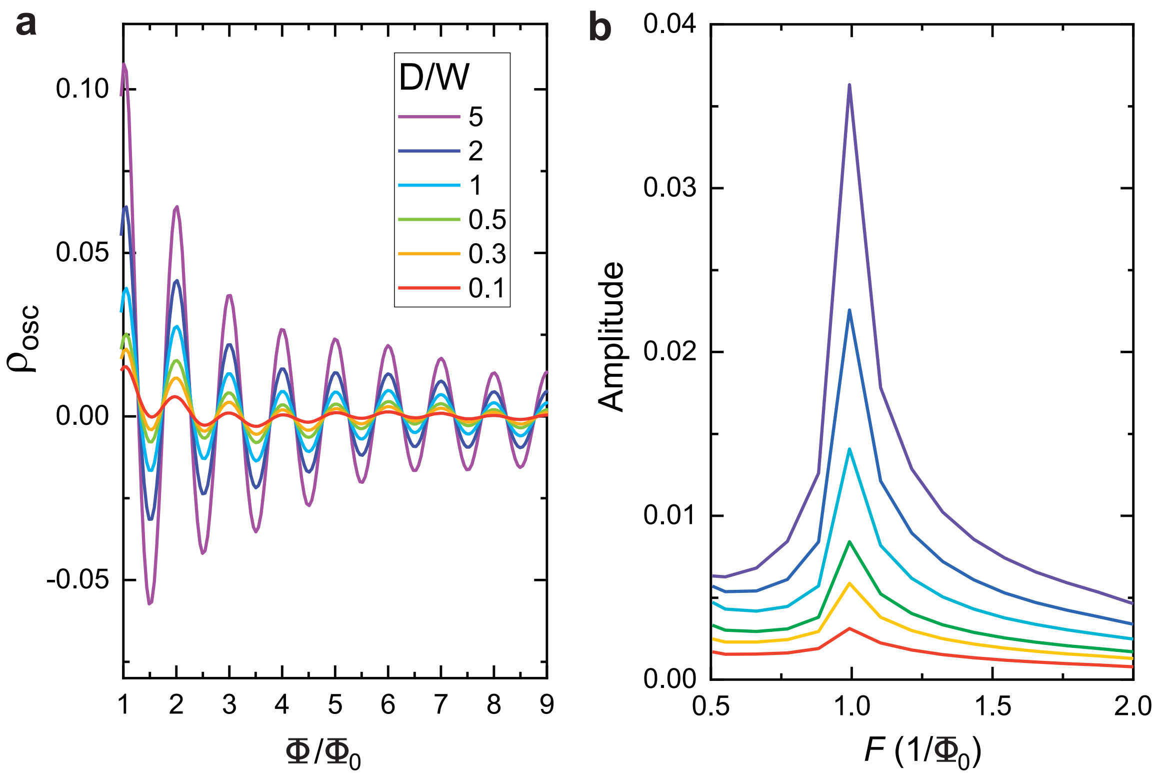

Fig. S 11. **Thickness-scaling of Bloch-Lorentz oscillation amplitude.** (a) Calculated Bloch-Lorentz oscillations at various depth-to-width ratios. (b) FFT analysis spectrum reveals a clear decrease in oscillation amplitude with decreasing depth-to-width ratio.

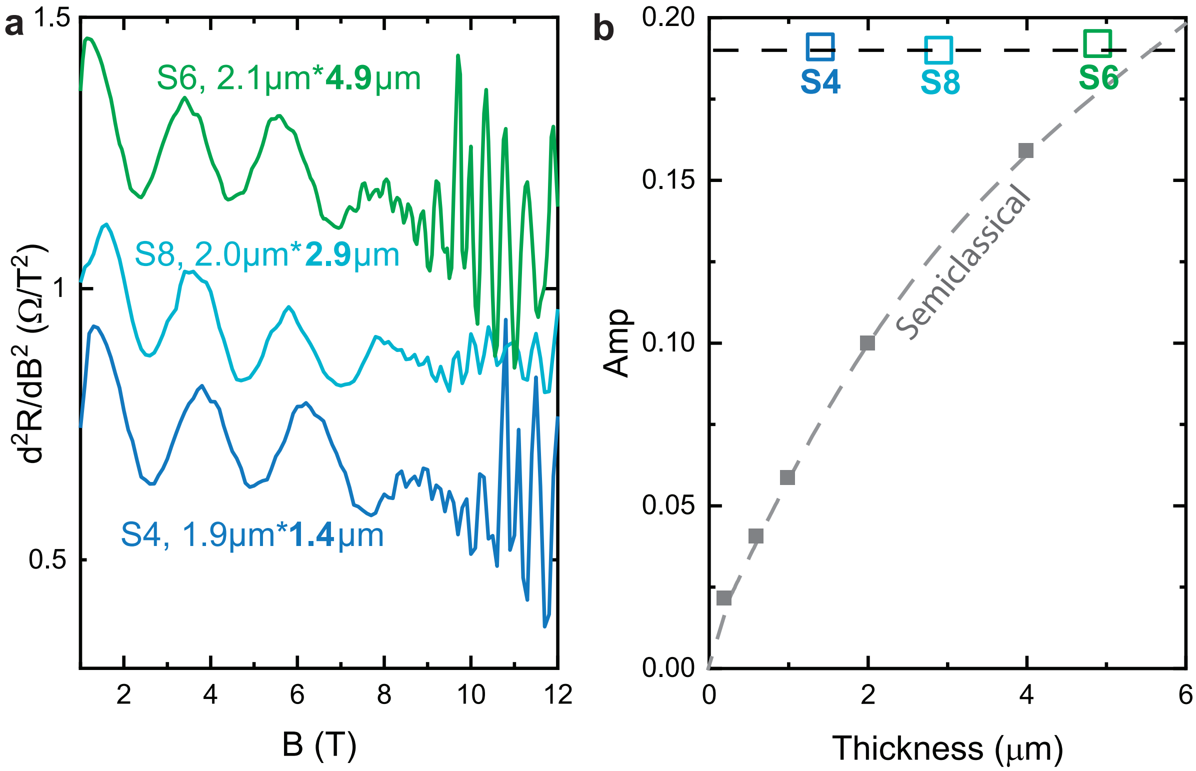

Fig. S 12. **Scaling of oscillation amplitude versus sample thickness.** (a) h/e oscillations observed among various devices with similar widths yet different depths. The oscillation amplitude remains nearly unchanged among different devices despite the substantial variation in depth. This robustness of amplitude against decreasing depth, as shown in (b), is in stark contrast with the semiclassical prediction, which again provides key evidence for quantum transport of collective electrons beyond semiclassical descriptions.

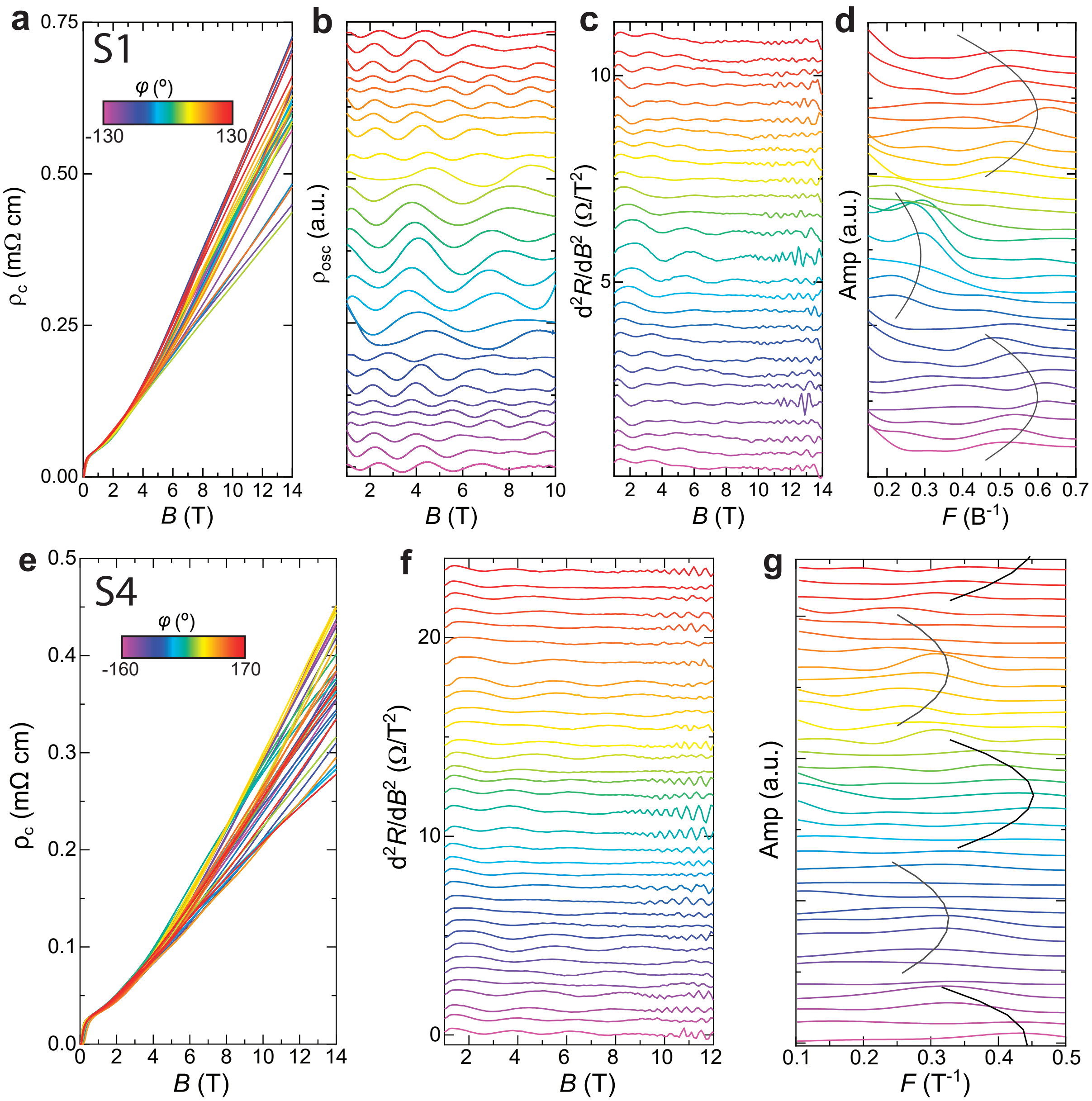

Fig. S 13. **In-plane angle-dependent h/e oscillations of S1 and S4.** (a) Angular dependence of magnetoresistance up to 14T. The measurements are conducted with 10° steps from -130° to 130°. (b) and (c) display the h/e oscillations extracted from polynomial background subtraction and second derivative, respectively. (d) FFT spectrum of h/e oscillations at different angles. The black line indicates the guideline for identified oscillation frequency peaks. (e) Angle-dependent magnetoresistance of S4 measured with a rotation from -160° to 170° in 10° steps. (f) Second derivative of magnetoresistance at all angles. (g) FFT spectrum of the oscillations at various angles.

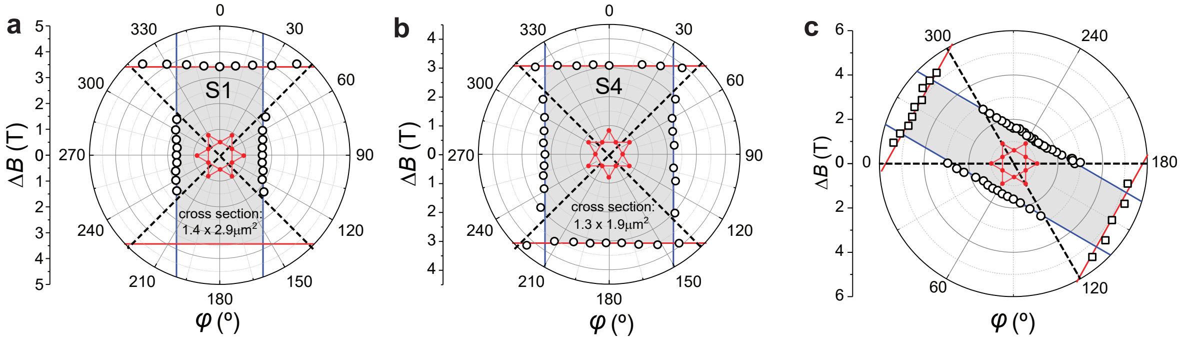

Fig. S 14. **Angular dependence of oscillation periods in device S1, S4, and tilt-cut S3.** Polar plot of oscillation period consistently demonstrates the switching behavior. The consistent observation of such behavior in three devices of distinct geometries further emphasizes that the switching has no correspondence to the sample's aspect ratio.

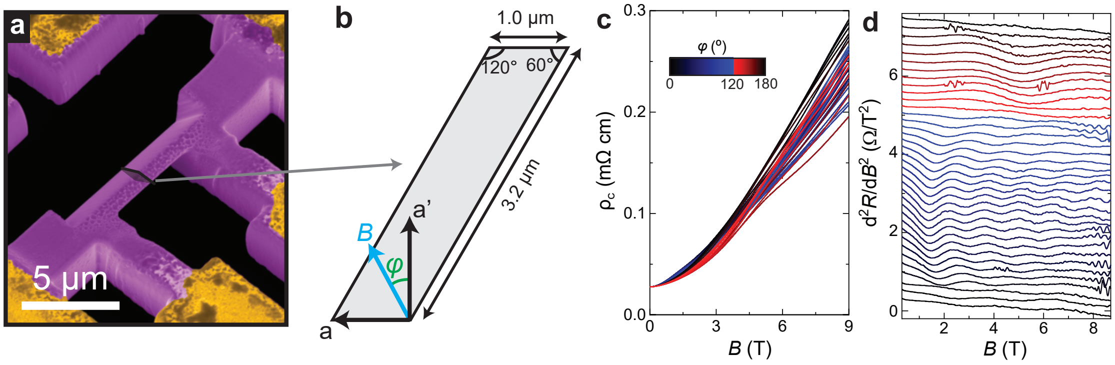

Fig. S 15. **In-plane angle-dependent h/e oscillations of tilt-cut sample.** (a) SEM image of the tilt-cut device S3 at 30 degrees. (b) Illustration of device geometry. (c) Angle-dependent magnetoresistance measured with a rotation from 0° to 180° in 5° steps. (d) Second derivative of magnetoresistance at all angles.

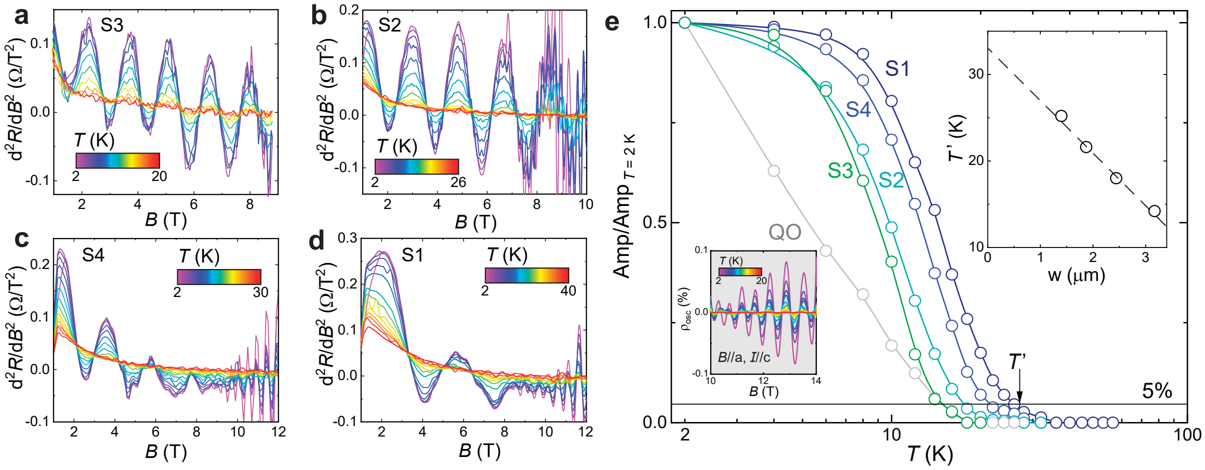

Fig. S 16. **Temperature dependence of h/e oscillation amplitude.** (a)-(d) display the temperature dependence of h/e oscillations in S3, S2, S4, S1, respectively. All measurements are conducted from 2 K to elevated temperatures in 2 K steps. (e) Temperature dependencies of oscillation amplitude across various devices and their comparison to quantum oscillations. The left-hand inset presents the temperature-dependent quantum oscillations measured with current and magnetic fields along the c- and a-direction. The right-hand inset displays the interpolation of T' at zero sample width by linearly extrapolating the temperature where the oscillation amplitude reaches 5% of the value at 2 K. This yields a $T' \approx 33$ K, which is almost identical to T' determined via other experimental results[5,12].

[1] F. H. Yu, T. Wu, Z. Y. Wang, B. Lei, W. Z. Zhuo, J. J. Ying, and X. H. Chen, Phys. Rev. B **104**, L041103 (2021).

[2] P. J. Moll, P. Kushwaha, N. Nandi, B. Schmidt, and A. P. Mackenzie, Science **351**, 1061 (2016).

[3] C. Guo, A. Alexandradinata, C. Putzke, A. Estry, T. Tu, N. Kumar, F.-R. Fan, S. Zhang, Q. Wu, O. V. Yazyev, et al., Nature Communications **12**, 6213 (2021).

[4] C. Putzke, M. D. Bachmann, P. McGuinness, E. Zhakina, V. Sunko, M. Konczykowski, T. Oka, R. Moessner, A. Stern, M. König, et al., Science **368**, 1234 (2020).

[5] C. Guo, C. Putzke, S. Konyzheva, X. Huang, M. Gutierrez-Amigo, I. Errea, D. Chen, M. G. Vergniory, C. Felser, M. H. Fischer, T. Neupert, and P. J. W. Moll, Nature **611**, 461 (2022).

[6] K. Vilkelis, L. Wang, and A. R. Akhmerov, SciPost Physics **15**, 019 (2023).

[7] K. Wang, C. Guo, P. J. Moll, and T. Holder, arXiv preprint arXiv:2409.16088 (2024).

[8] H. Zhao, H. Li, B. R. Ortiz, S. M. Teicher, T. Park, M. Ye, Z. Wang, L. Balents, S. D. Wilson, and I. Zeljkovic, Nature **599**, 216 (2021).

[9] L. Yu, C. Wang, Y. Zhang, M. Sander, S. Ni, Z. Lu, S. Ma, Z. Wang, Z. Zhao, H. Chen, K. Jiang, Y. Zhang, H. Yang, F. Zhou, X. Dong, S. L. Johnson, M. J. Graf, J. Hu, H.-J. Gao, and Z. Zhao, arXiv:2107.10714 (2021).

[10] L. Nie, K. Sun, W. Ma, D. Song, L. Zheng, Z. Liang, P. Wu, F. Yu, J. Li, M. Shan, D. Zhao, S. Li, B. Kang, Z. Wu, Y. Zhou, K. Liu, Z. Xiang, J. Ying, Z. Wang, T. Wu, and X. Chen, Nature **604**, 59 (2022).

[11] D. Chen, B. He, M. Yao, Y. Pan, H. Lin, W. Schnelle, Y. Sun, J. Gooth, L. Taillefer, and C. Felser, arXiv:2110.13085 (2021).

[12] C. Guo, G. Wagner, C. Putzke, D. Chen, K. Wang, L. Zhang, M. Gutierrez-Amigo, I. Errea, M. G. Vergniory, C. Felser, et al., Nature Physics **20**, 579 (2024).

[13] Y. Xiang, Q. Li, Y. Li, W. Xie, H. Yang, Z. Wang, Y. Yao, and H.-H. Wen, Nat. Commun. **12**, 6727 (2021).

[14] X. Wei, C. Tian, H. Cui, Y. Zhai, Y. Li, S. Liu, Y. Song, Y. Feng, M. Huang, Z. Wang, et al., Nature Communications **15**, 5038 (2024).

[15] Y.-X. Jiang, J.-X. Yin, M. M. Denner, N. Shumiya, B. R. Ortiz, G. Xu, Z. Guguchia, J. He, M. S. Hossain, X. Liu, J. Ruff, L. Kautzsch, S. S. Zhang, G. Chang, I. Belopolski, Q. Zhang, T. A. Cochran,

D. Multer, M. Litskevich, Z.-J. Cheng, X. P. Yang, Z. Wang, R. Thomale, T. Neupert, S. D. Wilson, and M. Z. Hasan, Nature Materials **20**, 1353 (2021).

[16] Z. Liang, X. Hou, F. Zhang, W. Ma, P. Wu, Z. Zhang, F. Yu, J.-J. Ying, K. Jiang, L. Shan, Z. Wang, and X. Chen, Phys. Rev. X **11**, 031026 (2021).

[17] H. Usui, M. Ochi, S. Kitamura, T. Oka, D. Ogura, H. Rosner, M. W. Haverkort, V. Sunko, P. D. King, A. P. Mackenzie, et al., Physical Review Materials **3**, 045002 (2019).

[18] H. Deng, H. Qin, G. Liu, T. Yang, R. Fu, Z. Zhang, X. Wu, Z. Wang, Y. Shi, J. Liu, H. Liu, X.-Y. Yan, W. Song, X. Xu, Y. Zhao, M. Yi, G. Xu, H. Hohmann, S. C. Holbæk, M. Dürrnagel, S. Zhou, G. Chang, Y. Yao, Q. Wang, Z. Guguchia, T. Neupert, R. Thomale, M. H. Fischer, and J.-X. Yin, Nature **632**, 775 (2024).

[19] M. S. Hossain, Q. Zhang, E. S. Choi, D. Ratkovski, B. Lüscher, Y. Li, Y.-X. Jiang, M. Litskevich, Z.-J. Cheng, J.-X. Yin, et al., Nature Physics , 1 (2025).

[20] B. R. Ortiz, S. M. Teicher, Y. Hu, J. L. Zuo, P. M. Sarte, E. C. Schueller, A. M. Abeykoon, M. J. Krogstad, S. Rosenkranz, R. Osborn, R. Seshadri, L. Balents, J. He, and S. D. Wilson, Phys. Rev. Lett. **125**, 247002 (2020).
